# Comparative Evaluation of Medial Septal Fat Excision During Infrabrow Blepharoplasty for Medial Upper Eyelid Fullness: A Retrospective Study

**DOI:** 10.3390/jcm15103637

**Published:** 2026-05-09

**Authors:** Seok Beom Lim, Marine Jung, Jong Hyup Kim, In Chang Koh, Soo Yeon Lim, Wan Cheol Ryu

**Affiliations:** 1 Department of Plastic and Reconstructive Surgery, Myunggok Medical Research Institute, Konyang University Hospital, Konyang University College of Medicine, Daejeon 35365, Republic of Korea; eric615@daum.net (S.B.L.); kimjh9437@naver.com (J.H.K.); ihns@naver.com (I.C.K.); 2 Department of Health & Rehabilitation Sciences, Boston University, Boston, MA 02215, USA; marine@bu.edu; 3 Department of Plastic and Reconstructive Surgery, Jenith Hospital, Ulsan 44703, Republic of Korea; ertgsd@naver.com

**Keywords:** infrabrow blepharoplasty, medial upper eyelid fullness, septal fat excision, dermatochalasis, eyelid surgery, cosmetic surgery, retrospective study

## Abstract

**Background/Objectives**: Medial upper eyelid fullness resulting from septal fat prolapse during infrabrow blepharoplasty has not been consistently addressed. However, the potential benefit of medial septal fat excision in enhancing the medial contour remains unclear. This study aimed to evaluate the efficacy and safety of medial septal fat excision during infrabrow blepharoplasty. **Methods**: This retrospective comparative cohort study included 488 patients who underwent infrabrow blepharoplasty with at least 6 months of follow-up. The patients were divided into the excision (*n* = 358) and non-excision (*n* = 130) groups based on the medial septal fat excision status. Medial fullness was graded using a standardized 4-point photographic scale. The primary outcome was the change in medial fullness grade (Δ). Analyses were performed at the patient level, selecting the eye with higher preoperative grade. Analysis of covariance was used to adjust for baseline differences. A subgroup analysis was performed for patients with mild baseline fullness (grades 1–2). **Results**: The excision group demonstrated significantly greater improvement in medial fullness. After adjustment for baseline differences, postoperative scores were significantly lower in the excision group, with an adjusted mean difference of −0.395. Subgroup analysis confirmed superior improvement in the excision subgroup. The complication rates were low and comparable between the groups (15.4% vs. 10.0%), with no increase in major adverse events. **Conclusions**: Medial septal fat excision during infrabrow blepharoplasty significantly enhances medial upper eyelid contour without increasing complication rates. This approach is a safe and effective adjunct for addressing medial fat bulging.

## 1. Introduction

Infrabrow blepharoplasty is commonly performed in Asian populations to correct age-related changes in the upper eyelids, such as dermatochalasis and lateral hooding [1,2,3,4,5]. However, this procedure alone may not sufficiently address medial upper eyelid fullness, which primarily results from medial orbital fat herniation.

Medial upper eyelid fullness contributes to a fatigued or aged appearance [6]. It is typically associated with protrusion of orbital fat through a weakened orbital septum [6,7,8]. Although conventional upper blepharoplasty often includes fat excision or repositioning of orbital fat, the infrabrow approach rarely involves the direct management of medial fat, potentially leading to persistent fullness postoperatively [9,10].

In this study, medial septal fat excision was performed via an infrabrow incision, enabling direct reduction in medial fat bulging without requiring additional upper eyelid incision [11]. However, the clinical benefits of this adjunctive procedure have not yet been clearly established.

Previous studies of infrabrow blepharoplasty have primarily focused on surgical techniques or subjective patient satisfaction, with limited objective evaluations of medial upper eyelid fullness [1,2,7]. Furthermore, comparative analyses evaluating outcomes with and without medial fat excision remain limited.

Despite the widespread use of infrabrow blepharoplasty, optimal management of medial upper eyelid fullness remains controversial, particularly in Asian populations. While several surgical modifications have been proposed, objective evaluation of medial contour and direct comparison between different approaches remain limited. Therefore, further investigation is needed to clarify the clinical benefit of adjunctive techniques such as medial septal fat excision.

Accordingly, this study aimed to evaluate the effectiveness and safety of medial septal fat excision during infrabrow blepharoplasty using a retrospective comparative cohort design. Considering the lack of a standardized grading system for medial upper eyelid fullness, a photographic grading scale was developed and applied. Outcomes were then compared between the excision and non-excision groups, with adjustments made for baseline differences.

## 2. Materials and Methods

### 2.1. Study Design and Patient Selection

This retrospective, comparative cohort study included patients who underwent infrabrow blepharoplasty at a single institution between January 2018 and December 2023. This study was conducted in accordance with the Declaration of Helsinki and approved by the Public Institutional Review Board of the Ministry of Health and Welfare (approval no. P01-202505-01-046, approval date: 21 May 2025). Written informed consent was obtained from the patients for the publication of this article.

Patients who (1) underwent infrabrow blepharoplasty during the study period, (2) had available standardized preoperative and postoperative photographs, and (3) were followed for at least 6 months were analyzed.

Conversely, patients who (1) had previously undergone upper eyelid surgery; (2) underwent concomitant procedures that could affect the upper eyelid contour, including double eyelid surgery, ptosis repair, or forehead lifting; (3) presented with traumatic or reconstructive cases; and (4) lacked adequate photographic documentation were excluded.

Patients were divided into two groups: excision and non-excision, according to whether medial septal fat excision was performed during infrabrow blepharoplasty.

All procedures were performed by multiple surgeons using a standardized surgical technique to ensure consistency across cases. Standardized photographs were obtained under consistent conditions, including fixed camera distance, lighting, and patient positioning.

### 2.2. Surgical Technique

Intravenous anesthesia was administered using propofol. A local anesthetic solution (10 cc of 1% lidocaine with epinephrine 1:200,000) was injected along the incision lines with a 30-gauge needle. An infrabrow incision was made along the inferior border of the eyebrow, and redundant skin and subcutaneous tissues were excised.

**Figure 1:**
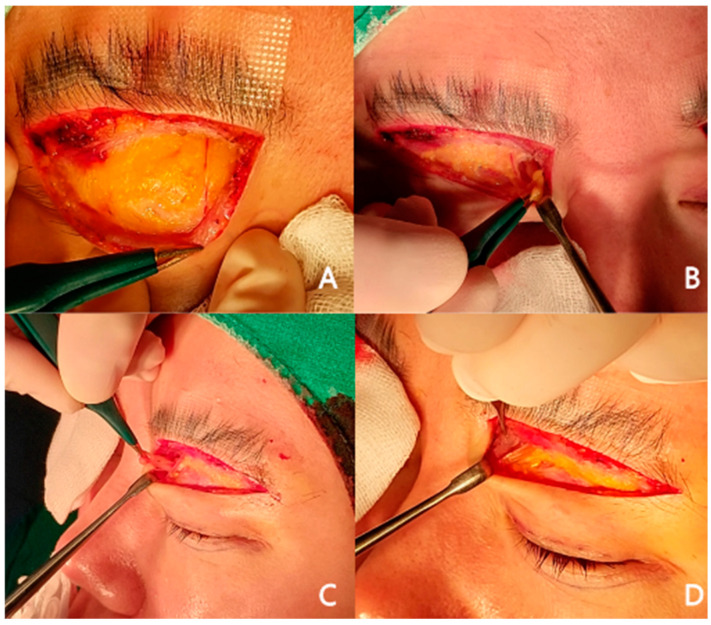
(**A**) The supratrochlear nerve branch is visualized during excision of the OOM and ROOF. (**B**) During medial orbital fat excision, careful dissection is necessary to avoid nerve injury. (**C**) Following ROOF excision and medial dissection, blunt retraction of the medial side facilitates herniation of bulging septal fat, which is then excised through a minimally sized window. (**D**) To prevent postoperative hollowness, careful assessment ensures that only an appropriate amount of fat is removed.

In the excision group, a strip of the orbicularis oculi muscle was excised, followed by the removal of the retro-orbicularis oculi fat to expose the orbital septum. The septum was carefully opened, medial orbital fat was identified, and protruding fat was excised. The extent of medial fat excision was determined intraoperatively based on the degree of medial fullness, with excision limited to the protruding portion of septal fat exposed after opening the septum to avoid over-resection. Hemostasis was achieved, and the wound was closed in layers. In the non-excision group, infrabrow blepharoplasty was performed without excision of the orbicularis oculi muscle, opening of the septum, or removal of orbital fat (Figure 1).

### 2.3. Medial Fullness Grading System

The medial upper eyelid region was defined as the area extending from the medial canthus to the medial third of the eyelid.

**Figure 2:**
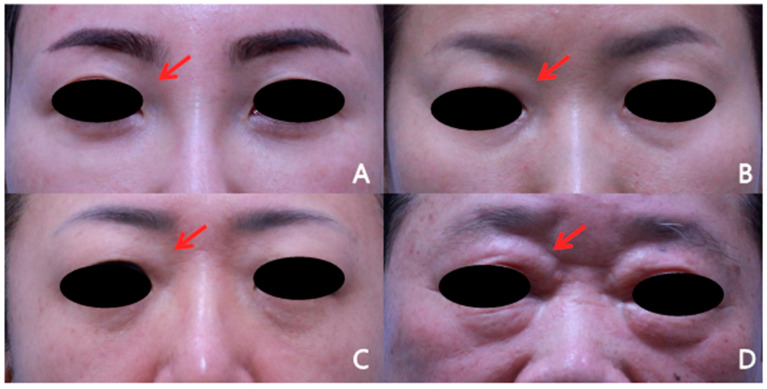
Representative examples of the proposed medial upper eyelid fullness grading scale. Standardized photographs illustrate (**A**) Grade 1 (minimal or no visible medial convexity), (**B**) Grade 2 (mild convexity without clear anterior protrusion), (**C**) Grade 3 (definite protrusion with a visible contour change), and (**D**) Grade 4 (severe bulging with obvious anterior projection). Arrows indicate the medial upper eyelid area.

Due to the absence of an established grading system for medial upper eyelid fullness, a novel 4-point photographic grading scale was developed and applied in this study. Medial upper eyelid fullness was assessed using standardized preoperative and postoperative frontal photographs obtained in the primary gaze with the eyebrows in a relaxed position (Figure 2).

The grading criteria were defined as follows:

- Grade 1: Minimal or no visible medial convexity;
- Grade 2: Mild convexity without clear anterior protrusion;
- Grade 3: Definite protrusion with visible contour change;
- Grade 4: Severe bulging with obvious anterior projection.

For analysis, the eye with a higher preoperative grade was selected. When both eyes exhibited identical grades, the right eye was selected based on a predefined rule.

To assess the reliability of the grading system, inter-rater reliability was evaluated using a randomly selected subset of patients. Three independent plastic surgeons, blinded to the clinical information, graded the medial fullness. Inter-rater reliability demonstrated good agreement (intraclass correlation coefficient [ICC] = 0.821, 95% confidence interval [CI] = 0.761–0.869, *p* < 0.001).

### 2.4. Outcome Measures

The primary outcome was the change in medial fullness grade (Δ), defined as the difference between preoperative and postoperative grades.

Secondary outcomes included postoperative complication rates, such as hollowness, hematoma, hypertrophic scarring, numbness, stitch abscess, and the need for revision surgery.

### 2.5. Statistical Analysis

Continuous variables are expressed as the mean ± standard deviation, whereas categorical variables are expressed as frequencies and percentages.

Between-group comparisons of continuous variables, including baseline characteristics and changes in medial fullness (Δ), were conducted using independent *t*-tests. Within-group changes in medial fullness (preoperative vs. postoperative) were analyzed using paired *t*-tests. Categorical variables, including sex and overall complication rates, were compared using the chi-square test.

To adjust for baseline differences in medial fullness, an analysis of covariance (ANCOVA) was performed, with postoperative medial fullness grade as the dependent variable and preoperative grade as a covariate. Adjusted means and 95% CIs were calculated. A post hoc power analysis demonstrated that the study had sufficient statistical power (>99%) to detect the observed effect size. To further reduce selection bias, a subgroup analysis was performed in patients with mild baseline medial fullness (grades 1–2).

Statistical significance was set at *p* < 0.05. All statistical analyses were performed using IBM SPSS Statistics version 29 (IBM Corp., Armonk, NY, USA).

## 3. Results

### 3.1. Patient Characteristics

A total of 488 patients met the inclusion criteria, comprising 358 in the excision group and 130 in the non-excision group. The mean follow-up duration was comparable between the two groups (19.10 ± 17.54 months vs. 18.71 ± 20.60 months, *p* = 0.842).

No significant differences were observed between groups in terms of age or sex. However, the excision group demonstrated a significantly higher baseline medial fullness grade compared with the non-excision group (2.68 ± 0.87 vs. 1.41 ± 0.62, *p* < 0.001) (Table 1).

### 3.2. Reliability of the Grading System

Inter-rater reliability analysis demonstrated good agreement among the three evaluators, with an ICC of 0.821 (95% CI: 0.761–0.869, *p* < 0.001).

### 3.3. Overall Improvement in Medial Fullness

In the excision group, the medial fullness grade significantly decreased from 2.68 ± 0.87 preoperatively to 1.55 ± 0.65 postoperatively (*p* < 0.001).

**Figure 3:**
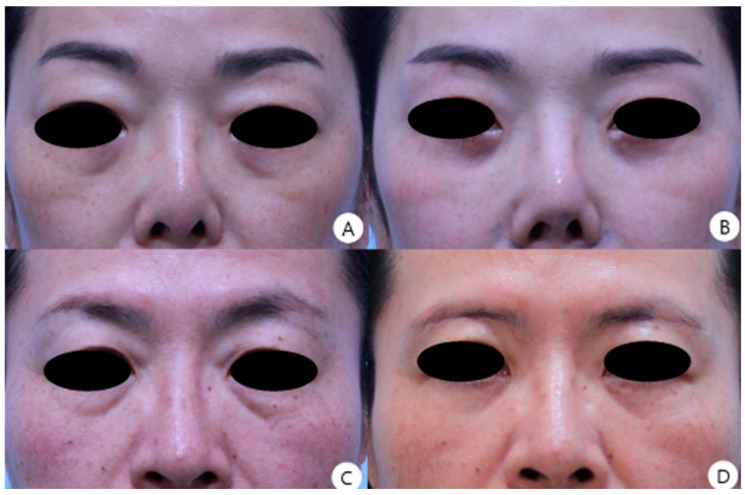
Preoperative and postoperative clinical photos. (**A**) A 50-year-old female patient underwent infrabrow blepharoplasty with medial septal fat excision with lower blepharoplasty to address lower eyelid dermatochalasis. (**B**) The 8-month postoperative view shows improvement in upper eyelid blepharochalasis and resolution of medial fat bulging. (**C**) A 52-year-old female patient underwent infrabrow blepharoplasty with medial septal fat excision with lower blepharoplasty to address lower eyelid dermatochalasis. (**D**) The 7-month postoperative view shows improvement in upper eyelid blepharochalasis, resolution of medial fat bulging.

In the non-excision group, no significant improvement was observed (1.41 ± 0.62 to 1.45 ± 0.57, *p* = 0.222). Representative examples are shown in Figure 3.

### 3.4. Comparative Analysis Between Groups

The mean reduction in medial fullness (Δ) was significantly greater in the excision group than in the non-excision group (1.11 ± 0.83 vs. −0.05 ± 0.43, *p* < 0.001).

After adjusting for baseline differences using ANCOVA, postoperative medial fullness scores remained significantly lower in the excision group (1.417 ± 0.031 vs. 1.813 ± 0.056, *p* < 0.001), with an adjusted mean difference of −0.395 (95% CI: −0.513 to −0.277). The effect size was 0.065 (partial η^2^) (Table 2).

### 3.5. Subgroup Analysis (Baseline Grades 1–2)

A subgroup analysis was performed in patients with mild baseline medial fullness (grades 1–2).

Within this subgroup, the excision group demonstrated significantly greater improvement compared with the control group (Δ: 0.55 ± 0.54 vs. −0.10 ± 0.37, *p* < 0.001).

Although baseline grades differed between the groups, adjustment using ANCOVA confirmed a significantly lower postoperative score in the excision group (1.173 ± 0.037 vs. 1.562 ± 0.044, *p* < 0.001), with an adjusted mean difference of −0.416 (95% CI: −0.540 to −0.291) (Table 3).

### 3.6. Complications

Complication rates are summarized in Table 4.

The overall complication rates were 15.4% and 10.0% in the excision and non-excision groups, respectively, with no significant intergroup difference (*p* = 0.214).

Hollowness was observed in 2.5% of patients in the excision group and was not observed in the non-excision group. Hematoma occurred in 2 (0.6%) cases in the excision group and none in the control group. No cases required revision surgery.

## 4. Discussion

This study evaluated the efficacy and safety of medial septal fat excision during infrabrow blepharoplasty using a comparative cohort design. The results demonstrated that medial septal fat excision led to significantly greater improvement in medial upper eyelid fullness compared with infrabrow blepharoplasty alone. This additional benefit was achieved without a significant increase in complication rates.

The present findings suggest that residual medial fullness after infrabrow blepharoplasty may be largely attributable to unaddressed medial fat protrusion. As the infrabrow approach primarily targets redundant skin, it may be insufficient to correct the medial contour when orbital fat contributes to fullness. In this context, direct management of medial septal fat appears to be an important factor in achieving optimal aesthetic outcomes.

A major strength of this study was the use of a comparative cohort design with a relatively large sample size. Previous studies on infrabrow blepharoplasty have primarily focused on surgical techniques or subjective patient satisfaction, with limited objective evaluation of medial upper eyelid fullness [12,13,14,15]. In contrast, the present study employed a standardized photographic grading system and directly compared the outcomes between the excision and non-excision groups.

Recent studies have highlighted evolving concepts in upper eyelid surgery, emphasizing volume preservation and redistribution in addition to fat excision [16,17]. Techniques such as medial fat reduction combined with pearl grafting have been proposed to improve eyelid contour by reducing medial prominence while augmenting central volume [16]. Furthermore, recent studies have incorporated objective imaging modalities, such as high-resolution ultrasonography, to quantitatively assess volumetric changes following fat repositioning and grafting procedures [17]. In contrast to these approaches, the present study demonstrates that selective excision of medial septal fat alone can provide significant improvement in medial upper eyelid fullness when appropriately applied.

Baseline medial fullness was significantly higher in the excision group, reflecting real-world surgical decision making. To account for potential selection bias, a subgroup analysis was performed in patients with mild baseline medial fullness (grades 1–2). Although the baseline grades were not identical between the groups, the superiority of the excision group persisted after adjustment using ANCOVA. These findings suggest that the observed effects cannot be solely attributed to baseline differences.

Another key strength of this study is the implementation of a standardized grading system for medial fullness. Given the lack of an established grading system for medial upper eyelid fullness, a novel grading system was developed. Inter-rater reliability, assessed by three independent observers, demonstrated good reproducibility (ICC = 0.821) [18].

From a safety perspective, concerns have been raised regarding postoperative hollowness due to over-resection and the potential for increased complications associated with additional manipulations. However, in the present study, the incidence of hollowness was low, and the overall complication rates did not differ significantly between the excision and non-excision groups. These findings indicate that medial septal fat excision can be safely performed without a significant increase in complication rates.

This study has some limitations. First, its retrospective design introduces the potential of selection bias. As it relied on existing clinical records and many patients had a follow-up period of less than 6 months, a substantial proportion were excluded due to insufficient follow-up, which may limit generalizability. Additionally, patient-reported outcomes, such as subjective satisfaction, were not assessed owing to the retrospective nature of the study. Future prospective studies incorporating validated patient-reported outcome measures and longer standardized follow-up intervals are warranted. Second, the grading system used in this study was not externally validated. Although the inter-rater reliability was good, the photo-based scoring remained partially subjective. Future studies incorporating three-dimensional imaging would be beneficial for providing a more objective evaluation of medial upper eyelid fullness. Third, this study was conducted at a single center, which may further limit the generalizability of the findings.

Despite these limitations, this study included a relatively large cohort with standardized follow-up and objective outcome assessments. These findings suggest that medial septal fat excision is a safe and effective adjunctive procedure during infrabrow blepharoplasty and provide practical guidance for surgical decision making, indicating that selective fat excision may enhance aesthetic outcomes in appropriately selected patients.

## Figures and Tables

**Table 1:** Demographic characteristics of patients.

Variable	Experimental (*n* = 358)	Comparative (*n* = 130)	*p*-Value
Sex (M/F)	63/295	17/113	0.248
Age (years)	54.64 ± 9.16	53.08 ± 8.34	0.089
Follow-up (months)	19.10 ± 17.54	18.71 ± 20.60	0.842
Preoperative grade	2.68 ± 0.87	1.41 ± 0.62	<0.001

**Table 2:** Comparison of preoperative, postoperative, and adjusted postoperative grades between the experimental group and comparative group.

Variable	Experimental (*n* = 358)	Comparative (*n* = 130)	*p*-Value
Preoperative grade	2.68 ± 0.87	1.41 ± 0.62	<0.001
Postoperative grade	1.55 ± 0.65	1.45 ± 0.57	0.087
Change (Δ)	1.11 ± 0.83	−0.05 ± 0.43	<0.001
Adjusted postoperative grade	1.417 ± 0.031	1.813 ± 0.056	<0.001
Adjusted mean difference (95% CI)	−0.395 (−0.513 to −0.277)	-	-
F-value	33.499	-	-
Effect size (partial η^2^)	0.065	-	-

**Table 3:** Comparison of preoperative, postoperative grade, and adjusted postoperative grades between the experimental and comparative subgroups.

Variable	Experimental-Subgroup (*n* = 157)	Comparative-Subgroup (*n* = 121)	*p*-Value
Preoperative grade	1.83 ± 0.38	1.29 ± 0.46	<0.001
Postoperative grade	1.28 ± 0.48	1.39 ± 0.52	0.043
Change (Δ)	0.55 ± 0.54	−0.10 ± 0.37	<0.001
Adjusted postoperative grade	1.173 ± 0.037	1.562 ± 0.044	<0.001
Adjusted mean difference (95% CI)	−0.416 (−0.540 to −0.291)	-	-
F-value	43.169	-	-
Effect size (partial η^2^)	0.136	-	-

**Table 4:** Complications of experimental group and comparative group.

Variable	Experimental (*n* = 358)	Comparative (*n* = 130)	*p* Value
Complications, n (%)	55 (15.4%)	13 (10.0%)	0.214
Scar	22 (6.1%)	7 (5.4%)	-
Dryness	11 (3.1%)	3 (2.3%)	-
Hollowness	9 (2.5%)	0 (0%)	-
Numbness	8 (2.2%)	2 (2.1%)	-
Stitch abscess	3 (0.8%)	1 (0.8%)	-
Hematoma	2 (0.6%)	0 (0%)	-
Revision surgery	0 (0%)	0 (0%)	-

## Data Availability

The raw data supporting the conclusions of this study are available from the corresponding author upon reasonable request.

## Abbreviations

The following abbreviations are used in this manuscript:

ANCOVA	Analysis of covariance
CI	Confidence interval
Δ	Change in medial fullness grade
ICC	Intraclass correlation coefficient
